# *Ramulus Mori* (Sangzhi) Alkaloids Alleviate High-Fat Diet-Induced Obesity and Nonalcoholic Fatty Liver Disease in Mice

**DOI:** 10.3390/antiox11050905

**Published:** 2022-05-05

**Authors:** Yan-Min Chen, Chun-Fang Lian, Qian-Wen Sun, Ting-Ting Wang, Yuan-Yuan Liu, Jun Ye, Li-Li Gao, Yan-Fang Yang, Shuai-Nan Liu, Zhu-Fang Shen, Yu-Ling Liu

**Affiliations:** 1 Institute of Materia Medica, Chinese Academy of Medical Sciences and Peking Union Medical College, Beijing 100050, China; chenyanmin@imm.ac.cn (Y.-M.C.); lianchunfang@imm.ac.cn (C.-F.L.); sunqianwen@imm.ac.cn (Q.-W.S.); yelinghao@imm.ac.cn (J.Y.); gaolili@imm.ac.cn (L.-L.G.); yangyf@imm.ac.cn (Y.-F.Y.); liusn@imm.ac.cn (S.-N.L.); shenzhf@imm.ac.cn (Z.-F.S.); 2 State Key Laboratory of Bioactive Substance and Function of Natural Medicines, Institute of Materia Medica, Chinese Academy of Medical Sciences & Peking Union Medical College, Beijing 100050, China; 3 Beijing Key Laboratory of Drug Delivery Technology and Novel Formulation, Institute of Materia Medica, Chinese Academy of Medical Sciences & Peking Union Medical College, Beijing 100050, China; 4 School of Pharmaceutical Sciences, Tsinghua University, Beijing 100084, China; tingtingwang@tsinghua.edu.cn (T.-T.W.); liuyysk@tsinghua.edu.cn (Y.-Y.L.)

**Keywords:** NAFLD, obesity, *Ramulus Mori* (Sangzhi) alkaloids, steatosis, oxidative stress, adiponectin

## Abstract

Nonalcoholic fatty liver disease (NAFLD), obesity, and type 2 diabetes mellitus (T2DM) have highly related mechanisms. *Ramulus Mori* (Sangzhi) alkaloids (SZ-A) from *Morus alba* L. were approved in 2020 for the treatment of T2DM. In this study, we examined the therapeutic effects and mechanism of SZ-A on obesity and NAFLD in mice. Mice (C57BL/6J) fed a high-fat diet (HFD) for 14 weeks were treated with SZ-A for another 6 weeks. HFD-induced weight gain was reduced by SZ-A in a dose-dependent manner. SZ-A treatment significantly stimulated adiponectin expression and secretion in adipose tissue and 3T3-L1 adipocytes. Additionally, SZ-A markedly reduced hepatic steatosis (triglyceride, total cholesterol) and expression of pro-inflammatory and pro-fibrotic genes. SZ-A regulated lipid metabolism and oxidative stress (malondialdehyde (MDA), superoxide dismutase (SOD), glutathione peroxidase (GPx), and glutathione (GSH)) in the liver. Palmitic acid-induced insulin resistance and lipid accumulation in HepG2 cells were also repressed by SZ-A. Collectively, SZ-A protected mice from HFD-induced NAFLD through an indirect effect of improved systemic metabolism reducing bodyweight, and a direct effect by enhancing the lipid metabolism of HepG2 cells. The weight-loss effect of SZ-A in mice was partly due to improved fatty oxidation instead of influencing food consumption.

## 1. Introduction

Non-alcoholic fatty liver disease (NAFLD) has emerged as the most common cause of chronic liver disease worldwide, affecting approximately 25% of the population [1]. The prevalence of NAFLD has increased with the growing obesity epidemic. NAFLD is considered a hepatic manifestation of metabolic syndrome, ranging from steatosis and non-alcoholic steatohepatitis (NASH) to fibrosis and cirrhosis [2]. Obesity and obesity-related metabolic diseases such as NAFLD, type 2 diabetes mellitus (T2DM), and cardiometabolic diseases share common pathophysiological mechanisms, including a disturbed gut microbiome, insulin resistance, and chronic inflammation [3,4]. Obesity results when unbalanced energy intake and expenditure causes excessive energy to be stored in adipose tissue as triglycerides. When storage capacity is overwhelmed, lipotoxic metabolic stress promotes chronic inflammation and insulin resistance. Inappropriate ectopic lipid accumulation in the liver, caused by insulin resistance, induces metabolic stress, mitochondrial dysfunction, and endoplasmic reticulum (ER) stress, which leads to cell death and metabolic inflammation [5]. Hepatic fibrosis is thought to result from prolonged inflammation and hepatic stellate cell (HSC) activation [6,7].

Disturbed lipid homeostasis and metabolic stress-induced damage are the key pathogenetic features of NAFLD [8,9]. AMP-activated protein kinase (AMPK), the guardian of metabolism, phosphorylates targets involved in lipid metabolism, protein metabolism, glucose metabolism, and mitochondrial homeostasis. Acetyl-CoA carboxylase (ACC) phosphorylation by activated AMPK regulates fatty acid synthesis and lipid β-oxidation. Peroxisome proliferator-activated receptor-γ co-activator 1α (PGC1α) is another downstream effector that regulates mitochondrial biogenesis [10]. When the balance between lipid production and breakdown is disturbed, free fatty acids (FFA) accumulated in the liver may be used as substrates for the production of lipotoxic species, which induce ER stress, mitochondrial dysfunction, oxidative stress, cell damage, and cytokine release. Mitochondria are a major cellular source of reactive oxygen species (ROS) [11]. Superoxide dismutase (SOD), glutathione peroxidase (GPx), and glutathione (GSH) are vital scavengers of ROS that relieve oxidative stress [12]. Excessive ROS induced by high fat diet (HFD) is harmful to hepatocytes, Kupffer cells, and hepatic stellate cells, which promotes the progression of NAFLD to NASH [13].

To date, no pharmacological therapies have been approved for NAFLD treatment. Given the complexity of NAFLD pathophysiology, agents targeting different molecular processes of hepatic metabolism, oxidative stress-induced cell death, inflammation, and fibrosis have been investigated in preclinical models and clinical trials [14].These agents act through direct effect in the liver and indirect effect by improving systemic metabolic indexes. A number of anti-diabetic agents, such as thiazolidinedione insulin sensitizers, glucagon-like peptide 1 (GLP1) receptor agonists, and sodium-glucose cotransporter-2 (SGLT-2) inhibitors, are studied in NAFLD clinical trials by increasing insulin sensitivity, reducing bodyweight, and improving systemic metabolism [4,15,16]. In addition, combination therapies with different targets represent a new approach to treat NAFLD because the therapeutic effects of monotherapy drugs with a single target are limited [17]. 

*Ramulus Mori* (Sangzhi) alkaloid (SZ-A) tablets, extracted from *Morus alba* L. (mulberry twig), were approved by the China National Medical Products Administration in 2020 for T2DM treatment in China (approval number Z20200002). The main components of SZ-A powder (SZ-A tablet materials) are alkaloids (50% or more by weight), including 1-deoxynojirimycin (DNJ), 1,4-dideoxy-1,4-imino-D-arabinitol (DAB), and fagomine (FA). Though the prophylactic effects of DNJ have been reported to protect against high fat diet-induced liver steatosis through the regulation of the gut microbiota [18] and mitochondrial biogenesis [19], the application of DNJ is restricted, as industrial scale production of DNJ is difficult [20]. Enzymatic studies and molecular docking revealed that SZ-A inhibits α-glucosidases, especially disaccharidases [21]. Chronic treatment with SZ-A protects mice from HFD-induced insulin resistance and modulates gut microbiota and gut barrier integrity in type 2 diabetic KKAy mice [22]. In addition, the anti-inflammatory effects of SZ-A were also reported to result from blocking the activation of the p38 MAPK, ERK, and JNK signaling pathways in macrophages [23]. SZ-A exhibited hypoglycemic effects in the treatment of T2DM with fewer gastrointestinal side effects in a multicenter, randomized, double-blind clinical trial [24]. Tissue distribution analysis showed that the major alkaloids of SZ-A are well absorbed after oral administration and rapidly distributed to the liver and kidneys, in addition to the gastrointestinal tract [25], which revealed that SZ-A may have a direct effect on these tissues. 

In the present study, we investigated whether SZ-A mitigates obesity and NAFLD in HFD-fed mice by directly acting on the liver and adipose tissues. The direct effects and mechanisms of action of SZ-A and its major components on hepatocytes and adipocytes were studied in vitro. Our results demonstrate the potential of SZ-A as a natural anti-diabetic agent for the treatment of obesity and NAFLD in mice.

## 2. Materials and Methods

### 2.1. Chemical and Reagents

*Ramulus Mori* (Sangzhi) alkaloids (SZ-A) powder (lot number: J202107010, the total polyhydroxy alkaloids content in SZ-A powder is 60.62%, which includes 40.75% DNJ, 8.99% FA, and 8.59% DAB, as shown in Supplementary Figure S1) was kindly provided by Beijing Wehand-Bio Pharmaceutical Co., Ltd. (Beijing, China). 1-Deoxynojirimycin (DNJ, purity > 98.5%) was kindly provided by Institute of Materia Medica, Chinese Academy of Medical Sciences and Peking Union Medical College. Fagomine (FA, purity > 98.0%) was obtained from MedChemExpress (Shanghai, China). 1,4-dideoxy-1,4-imino-D-arabinitol (DAB, purity > 98.0%), dexamethasone (DEX), 3-isobutyl-1-methylxanthine (IBMX) and insulin were purchased from Sigma-Aldrich (St Louis, MO, USA). Palmitic acid (PA) was purchased from Beijing Solarbio Science & Technology Co., Ltd. (Beijing, China). β-actin, AMPK, p-AMPK, ACC, p-ACC, peroxisome proliferator activated receptor γ (PPARγ), uncoupling protein 2 (UCP2), and adiponectin primary antibodies were purchased from Cell Signaling Technology (Danvers, MA, USA, cat nos.: 4970, 2532, 2535, 3676, 11818, 9475, 2435, 89326, and 2789, respectively). Peroxisome proliferator-activated receptor-α (PPARα) and peroxisome proliferator-activated receptor-γ co-activator 1α (PGC1α) primary antibodies were obtained from Abcam (Cambridge, MA, USA, cat nos.: ab126285 and ab54481, respectively).

### 2.2. Experimental Animals and Treatment

All animal care and experimental procedures were approved by Beijing Laboratory Animal Research Center (ethical code: 2020001). Six-week-old male C57BL/6J mice were housed at 24 °C with a 12 h light/dark cycle. After 1 week of acclimatization to the environment, the mice were randomly divided into eight groups: a normal control group (NC; *n* = 10), an HFD control group by intragastrical administration of saline (HFD i.g.; *n* = 10), the SZ-A treatment groups by intragastrical administration (SZ-A 100; *n* = 10, SZ-A 200; *n* = 10, SZ-A 300; *n* = 10, SZ-A 400; *n* = 10), an HFD control group by peritoneal injection (HFD i.p. *n* = 10), and an SZ-A treatment group by peritoneal injection (SZ-A 200; *n* = 10). The mice in the NC group were given a standard diet, and the mice in other groups were fed with HFD (Research Diet, Cat No: D12492) for 14 weeks. Then the mice were given saline i.p., saline i.g., SZ-A 100 mg/kg/d i.g., SZ-A 300 mg/kg/d i.g., SZ-A 400 mg/kg/d i.g., and SZ-A 200 mg/kg/d i.p. for 6 weeks, respectively. Mice in the HFD and treatment groups were fed with HFD during the treatment. Bodyweights were measured weekly during the experimental period. Food intake was measured per cage. At the end of these experiments, mice were fasted overnight before being euthanized. Blood samples were collected and then kept at room temperature for half an hour before centrifugation at 3000 rpm for 15 min to obtain the serum samples. Tissues including liver, abdominal adipose tissue, perirenal fat, and subcutaneous fat were carefully collected, kept in liquid nitrogen, and then stored at −80 °C until analysis. Total body fat was measured using a Pharma Scan 70/16 US small animal MRI (Bruker, Ettlingen, Germany). 

### 2.3. Serum Biochemical Parameters

Serum total cholesterol (CHO), low-density lipoprotein cholesterol (LDL-C), C-reactive protein (CRP), high-density lipoprotein cholesterol (HDL-C), aspartate aminotransferase (AST), and alanine aminotransferase (ALT) levels were measured using automatic biochemistry analyzer (TOSHIBA TBA-40FR, Tokyo, Japan) with appropriate commercial assay kits from Biosino Biotechnology and Science Inc. (Beijing, China). Serum leptin level was determined using a mouse leptin ELISA kit (R&D Systems, Minneapolis, MN, USA) according to the manufacturer’s instruction. 

### 2.4. Hepatic Lipid and Antioxidant Measurement

The frozen liver tissues were homogenized in cold PBS (pH 7.4). The suspension was centrifuged at 3000× *g* for 5 min at 4 °C, and the supernatant was collected for the assay. Hepatic total cholesterol (TC) and triglyceride (TG) were determined by COD-PAP and GPO-PAP with the commercial kits (Nanjing Jiancheng Bioengineering Institute, Nanjing, China). To measure the hepatic lipid peroxidation, the liver malondialdehyde (MDA) concentrations were monitored using a thiobarbituric acid reactive substances (TBARS) assay kit (Applygen Technologies Inc., Beijing, China), and calculated using a standard curve. The reduced glutathione (GSH) content was determined using a thiol-specific reagent, dithionitrobenzoic acid, according to the manufacturer’s protocol (Nanjing Jiancheng Bioengineering Institute, Nanjing, China), and the adduct was measured at 420 nm. Glutathione peroxidase (GPx) activity was determined using a glutathione peroxidase assay kit (Nanjing Jiancheng Bioengineering Institute, Nanjing, China). In brief, the activity of GPx was measured using the assay kit based on the principle that oxidation of glutathione and H_2_O_2_ could be catalyzed by GPx to produce oxidized glutathione (GSSG) and H_2_O. According to the xanthine oxidase method, the activity of superoxide dismutase (SOD) was determined by superoxide dismutase activity colorimetric assay kit using water-soluble tetrazolium salt (Nanjing Jiancheng Bioengineering Institute, Nanjing, China). The concentration of proteins was estimated using a bicinchoninic acid (BCA) assay kit (Thermo Fisher Scientific, Waltham, MA, USA).

### 2.5. Histological Analysis

Fresh liver tissue, inguinal adipose tissue (iWAT), epididymal adipose tissue (eWAT), and brown adipose tissue (BAT) were fixed in 10% paraformaldehyde solution, embedded in paraffin wax, and cut at 5 μm for hematoxylin–eosin (H&E) staining. Liver sections of each animal with volumes of approximately 1 cm^3^ were excised and placed in a tissue container, which was then filled with Tissue-Tek OCT compound gel (Sakura Finetek, CA, USA) and frozen in liquid nitrogen. The frozen samples were cut into 7 μm slices and stained with Oil Red O solution (Baso diagnostics Inc, Zhuhai, China) for evaluation of fat accumulation. After removing the staining solution with 70% ethanol and distilled water, the sections were counterstained by hematoxylin for 2 min, washed in tap water for another 5 min, and mounted with glycerin. Images of stained liver sections were obtained with an Olympus microscope–camera system. In order to observe the ultrastructure of liver, a small part of liver tissue was fixed in glutaraldehyde solution (2.5%) for 4 h at 4 °C and post-fixed in 1% osmic acid with 0.1 M phosphate buffer for 1 h at 4 °C. After dehydration, infiltration, and embedding, liver tissue samples were cut and stained with uranyl acetate. Images were taken under a transmission electron microscope (TEM; Hitachi H7650, Tokyo, Japan) at 80 kV. 

### 2.6. Cell Culture and Differentiation

Human hepatocellular carcinoma cell line (HepG2 cells) and 3T3-L1 cell line were obtained from the Cell Resource Center, Peking Union Medical College (which is the headquarter of National Infrastructure of Cell Line Resource, NSTI). The HepG2 cells were cultured in DMEM supplemented with 10% FBS and 1% penicillin/streptomycin, and maintained in 100 mm dishes in the presence of 5% CO_2_ at 37 °C.

The PA-induced medium was prepared with serum-free DMEM using the methods described by Arwa et al. [26]. A 100 mmol/L stock solution of PA was prepared in ethanol and then conjugated to 1% fatty acid free-bovine serum albumin (BSA). The cells were treated with different concentrations of SZ-A by adding appropriate amounts of the PA/BSA conjugate to the cultured cells in DMEM media.

3T3-L1 pre-adipocytes were grown to confluence in DMEM containing 10% calf serum and 1% penicillin–streptomycin for 4 days. Then the cells were induced to differentiate with DMEM containing 10% (*v*/*v*) fetal bovine serum, 1 μmol/L DEX, 10 μg/mL insulin, and 0.5 mmol/L IBMX every 2 days for an additional 4 days. Cells were incubated with fresh DMEM containing 10% fetal bovine serum and 10 mg/mL insulin for another 2 days. Finally, the media were replaced with DMEM containing only 10% FBS and 1% penicillin–streptomycin for 2 days, after which the cells were used.

### 2.7. Cellular Lipid Analysis

HepG2 cells were plated in 6-well plates at 4 × 10^5^ cells per well for 24 h. SZ-A (12.5, 25, and 50 µg/mL), DNJ (40 µg/mL), FA (10 µg/mL), DAB (10 µg/mL), and 0.125 mmol/L PA were added in the culture medium at the same time for 24 h. The cells were washed with PBS and lysed in lysis buffer (1% Triton X-100 and 1% PMSF in PBS) for 4 h at 4 °C. The TG level of the cells was measured by GPO-PAP (Nanjing Jiancheng Bioengineering Institute, Nanjing, China). The protein concentrations were measured using BCA, and results were expressed as a fold change of intracellular TG level compared to the non-treated control.

### 2.8. Glucose Consumption and Glycogen Concentration Measurement

Glucose consumption and cellular glycogen concentration in HepG2 cells was assessed according to the previous report with a few modifications [27]. In short, cells cultured in six-well plates were treated with PA in the presence of SZ-A (12.5–50 µg/mL). After the treated HepG2 cells were incubated for 24 h, glucose content in the culture medium was measured by glucose oxidase using a glucose assay kit (Nanjing Jiancheng Bioengineering Institute, Nanjing, China). For the measurement of cellular glycogen, each group of cells was counted. The glycogen content of HepG2 cells was detected by anthrone−sulfuric acid using a glycogen assay kit (Nanjing Jiancheng Bioengineering Institute, Nanjing, China). Briefly, HepG2 cells were digested and centrifuged at 1000 rpm for 10 min, and the precipitates were transferred to glass tubes with 0.2 mL of alkaline liquor. Samples were incubated in boiling water for 10 min and diluted with distilled water. Thereafter, 1 mL of a colored-substrate solution and samples were incubated in boiling water for 5 min, according to the instructions. The absorbance values were measured at 620 nm by a microplate photometer (MultiSkan FC, Thermo Fisher Scientific, Rockville, MD, USA).

### 2.9. Supernent Adiponectin Measurement

The 3T3-L1 cells were seeded into 6-well plates at 2 × 10^4^ cells/mL and induced as described in cell culture and differentiation protocol. The differentiated 3T3-L1 preadipocytes were treated with different concentrations of SZ-A (100, 200 μg/mL), DNJ (40, 80 μg/mL), FA (10, 20 μg/mL), and DAB (10, 20 μg/mL) during differentiation. On the last day of the differentiation process, the media were collected, and concentrations of adiponectin were measured by ELISA (Elabscience, Wuhan, China).

### 2.10. Total RNA Preparation and Real-Time PCR Analysis

Total RNA was isolated from liver tissues using TRIzol^®^ (Invitrogen, Carlsbad, CA, USA). RNA was quantified by a Nano-300 Micro-Spectrophotometer (AllSheng, Hangzhou, China), and 1 µg RNA was reverse transcribed into cDNA using a reverse transcription system (Promega, Madison, WI, USA) according to the manufacturer’s protocol. The PCR amplification protocol consisted of 30 s at 95 °C, 10 s at 95 °C, and 30 s at 60 °C for 40 cycles. The purity of the PCR products was determined by melting curve analysis. The relative amount of each gene was calculated using 2^−ΔΔCT^. The level of transcripts was normalized, using β-actin as an internal standard. The primers used are provided in Table S1.

### 2.11. Western Blot

HepG2 cells, liver tissue, and iWAT were lysed in ice-cold lysis buffer containing protease inhibitor cocktail (Roche, Basel, Switzerland) and 1 mmol/L phenylmethanesulfonyl fluoride (PMSF) for 30 min, and were subjected to centrifugation at 10,000× *g* for 30 min at 4 °C. The protein concentration was detected by BCA assay. Equal amounts of protein samples were separated by 10% SDS-PAGE and transferred to polyvinylidene difluoride membranes. The membranes were blocked with blocking buffer (10% blocker milk in PBS) for 1 h and incubated with first antibodies including β-actin (1:1000), p-AMPK (1:1000), AMPK (1:1000), PPARα (1:1000), PPARγ (1:1000), PGC1α (1:1000), adiponectin (1:1000), and UCP2 (1:1000) overnight at 4 °C, then washed three times with Tris-buffered saline with Tween 20 (TBST) and incubated with the secondary antibody conjugated to anti-mouse (1:5000) or anti-rabbit (1:5000) HRP-conjugated secondary antibodies for 1 h. Bands were detected using enhanced chemiluminescence (ECL) and visualized by Tanon-4600SF chemiluminescence imager (Tanon Science & Technology Co., Ltd., Shanghai, China). 

### 2.12. RNA Sequencing and Data Analysis

RNA extraction and RNA-seq analysis were carried out by Novogene (Beijing, China). Briefly, total RNA from liver tissues was isolated using TRIzol reagent according to the manufacturer’s instructions, and RNA integrity was assessed using the RNA Nano 6000 Assay Kit of the Bioanalyzer2100 system (Agilent Technologies, Santa Clara, CA, USA). The mRNA was purified using poly-T oligo-attached magnetic beads. After fragmentation, libraries were constructed using NEBNext^®^ Ultra™ RNA Library Prep Kit for Illumina^®^ according to the manufacturer’s instructions. Library quality was checked using the Agilent Bioanalyzer 2100 system. After cluster generation, the library was sequenced using an Illumina Novaseq platform, and 150 bp paired-end reads were generated. RNA sequencing data have been submitted to the Gene Expression Omnibus (GEO) under accession number (GSE199105). 

High quality reads were aligned to the Ensemble mouse (mm10/GRCm38) reference genomes with HISAT2 (version 2.0.5) software. Differential expression analysis was performed using the DESeq2 R package (1.20.0). The resulting p-values were adjusted using Benjamini and Hochberg’s approach for controlling the false discovery rate. Transcripts with a Padj <0.05 and a fold change >1.2 were assigned as differential expression genes (DEGs). Gene Ontology (GO) and Kyoto Encyclopedia of Genes and Genomes (KEGG) enrichment analyses of DEGs were implemented by Novogene (Beijing, China).

### 2.13. Software and Statistical Analysis

Image J software (NIH, Bethesda, MD, USA) was used to quantify the relative expression of proteins and adipocyte area. Graphs were produced using Prism 8 software (GraphPad Software Inc., San Diego, CA, USA). Numeric results are expressed as the mean ± SEM. Two experimental groups were analyzed using unpaired two-tailed Student’s *t*-test. Multiple groups were compared using one-way or two-way analysis of variance (ANOVA) followed by the Tukey’s test depending on the experiments. Differences where *p* < 0.05 were considered statistically significant. 

## 3. Results

### 3.1. SZ-A Protects Mice from Hfd-Induced Obesity

**Figure 1:**
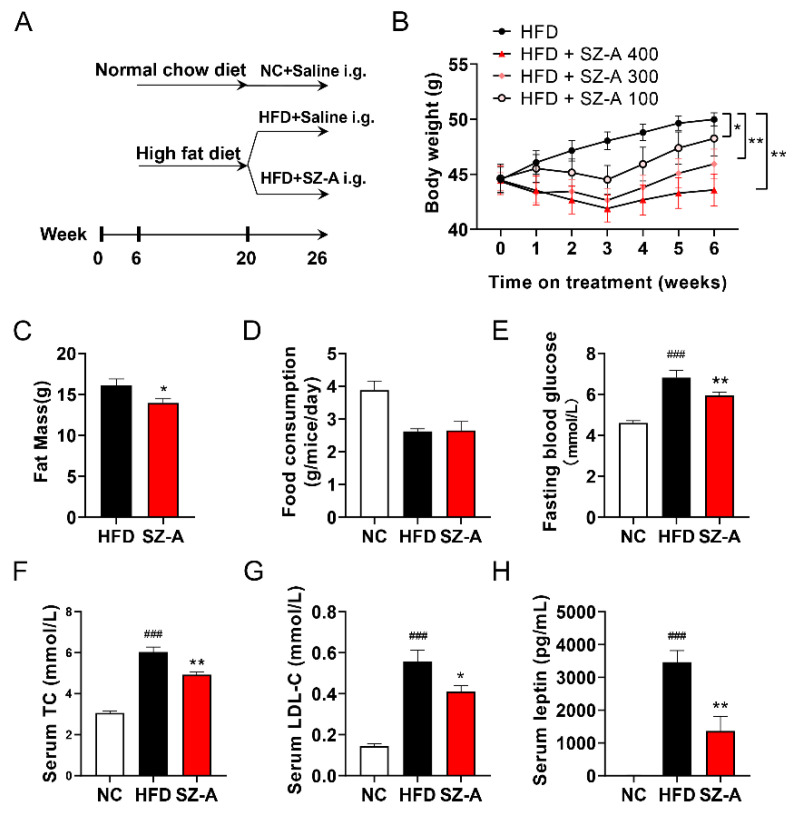
Effect of SZ-A on HFD-induced obesity. (**A**) Schematic diagram of the experimental procedure used to examine the protective effects of SZ-A on mice fed with HFD. HFD-fed mice were intragastrical administered (i.g.) saline or SZ-A (100, 300, and 400 mg/kg) once daily. (**B**) Bodyweight of mice treated with SZ-A (100, 300, and 400 mg/kg). (**C**) Total fat mass, (**D**) food consumption and (**E**) fasting blood glucose of mice. Effects of SZ-A (400 mg/kg) on serum levels of CHO (**F**), LDL-C (**G**), and leptin (**H**). Values are expressed as mean ± SEM (*n* = 10/group). ### *p* < 0.001 compared with the NC group (i.g.), and * *p* < 0.05, ** *p* < 0.01 compared with the HFD control group. NC, normal control; HFD; HFD, high-fat diet; SZ-A, *Ramulus Mori* (Sangzhi) alkaloids; TC, total cholesterol; LDL-C, low-density lipoprotein cholesterol.

To examine the therapeutic effects of SZ-A on obesity and NAFLD, 6-week-old C57 mice were fed an HFD for 14 weeks and administered SZ-A (i.g.) for an additional 6 weeks with continuous HFD feeding (Figure 1A). After feeding the HFD, the bodyweight of the HFD group was significantly increased compared to that of the normal chow diet-fed control group. SZ-A protected the mice from HFD-induced obesity in a dose-dependent manner, with SZ-A (400 mg/kg) showing the most significant effect (Figure 1B). Next, we measured the total body fat mass using magnetic resonance imaging (MRI). Remarkably, administration of 400 mg/kg SZ-A reduced total fat mass (Figure 1C). However, food consumption was not altered by SZ-A treatment compared to the HFD control group (Figure 1D). The increased fasting blood glucose levels in the HFD-fed mice were significantly reduced by SZ-A (Figure 1E). In addition, treatment with SZ-A markedly reduced total cholesterol (CHO) and low-density lipoprotein cholesterol (LDL-C) levels in HFD-fed mice (Figure 1F,G). Compared with the NC group, HFD mice developed leptin resistance, which led to a compensatory increase in serum leptin levels. SZ-A-treated mice showed reduced serum leptin levels, which reflected increased leptin sensitivity (Figure 1H). These results show that SZ-A protects mice from obesity and hyperlipidemia. 

### 3.2. SZ-A Stimulates Adiponectin Expression in Adipocytes

**Figure 2:**
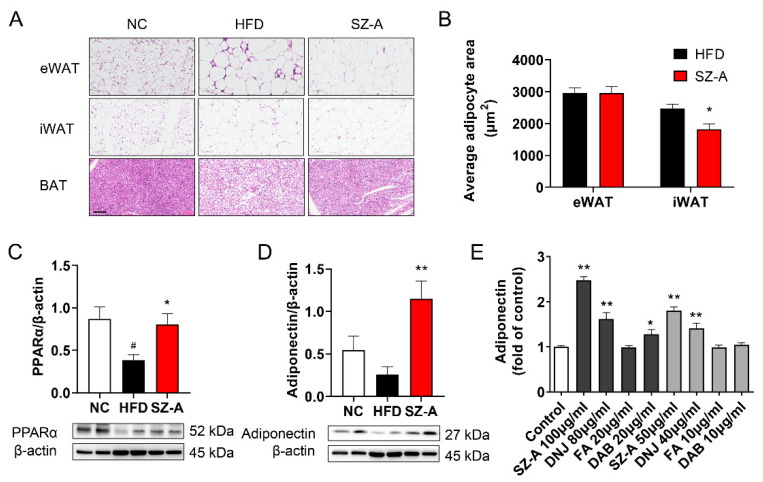
Effect of SZ-A on adipose tissues and adipocytes. (**A**) Representative H&E staining of dissected tissues. Scale bar, 100 μm. (**B**) Quantification of eWAT and iWAT adipocyte area. (**C**,**D**) Protein expressions of PPARα and adiponectin in iWAT (*n* = 4~6/group). Data are represented as mean ± SEM. # *p* < 0.05 compared with the NC mice (i.g.), and * *p* < 0.05, ** *p* < 0.01 compared with the HFD mice (i.g.). The protein amount of adiponectin (**E**) in the cell supernatant was determined by ELISA (*n* = 3). * *p* < 0.05, ** *p* < 0.01 compared with the corresponding control group. iWAT, inguinal adipose tissue; eWAT, epididymal adipose tissue; BAT, brown adipose tissue; NC, normal control; HFD; HFD, high-fat diet; SZ-A, *Ramulus Mori* (Sangzhi) alkaloids; PPARα, proliferator-activated receptor-α.

Next, we examined the direct effect of SZ-A on adipose tissue and adipocytes. Fewer immune cells infiltrated the eWAT of the SZ-A-treated mice (Figure 2A). BAT from SZ-A-treated mice was redder in color and showed less lipid accumulation than BAT from HFD control mice (Figure 2A). Adipocyte size analysis in iWAT revealed that the area of the enlarged adipocytes in HFD-fed mice was considerably reduced in SZ-A-treated mice, while the adipocyte area in eWAT was not altered by SZ-A (Figure 2B). PPARα is a nuclear transcription factor regulating lipid metabolism. Western blot showed that PPARα expression in iWAT was increased by SZ-A treatment (Figure 2C). Adiponectin is an important adipokine, secreted mainly by adipocytes, that exerts anti-inflammatory and insulin-sensitizing effects by binding to its receptors. The expression of adiponectin in iWAT was increased by SZ-A treatment (Figure 2D). Treatment of differentiating 3T3-L1 cells with SZ-A, DNJ, or DAB resulted in increased secretion of adiponectin into the culture medium (Figure 2E). These results indicate that SZ-A might exert its protective effects on obesity in mice by enhancing fatty acid oxidation. The weight loss effect of SZ-A improves systemic metabolism in mice.

### 3.3. SZ-A Alleviates Hepatic Steatosis and Injury in Mice

**Figure 3:**
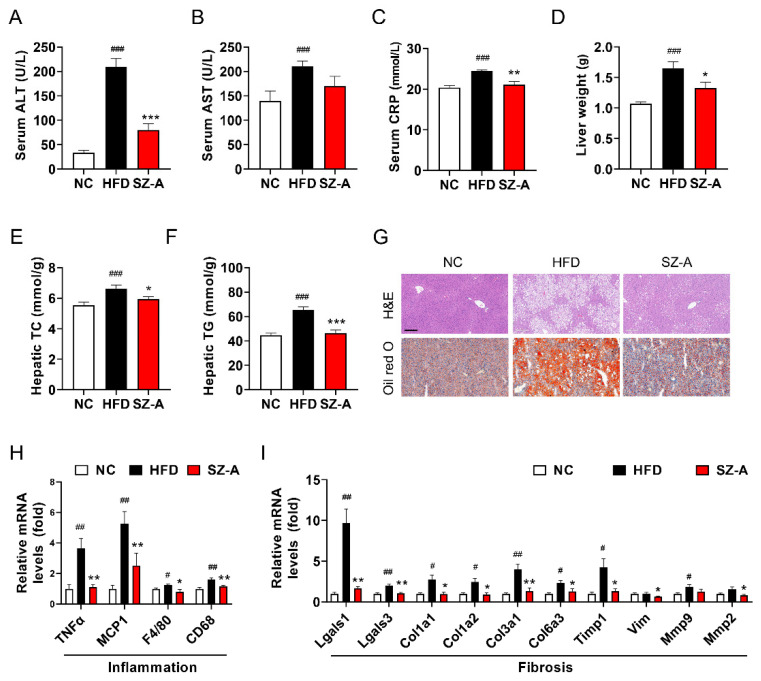
SZ-A alleviates hepatic steatosis and injury in mice fed with HFD. Effects of SZ-A on serum ALT (**A**), AST (**B**), and C-reaction protein (**C**) (*n* = 10/group). (**D**–**F**) Liver weight, hepatic triglyceride (TG), and total cholesterol (TC) in the three groups of mice (*n* = 10/group). (**G**) Histology of the livers stained with hematoxylin–eosin (H&E) and Oil Red O (scale bar, 200 μm). Relative mRNA expression levels of pro-inflammatory cytokines (**H**) and fibrosis (**I**) in livers (*n* = 6/group). Data are represented as mean ± SEM (*n* = 10/group). # *p* < 0.05, ## *p* < 0.01, ### *p* < 0.001 compared with the NC group (i.g.), and * *p* < 0.05, ** *p* < 0.01, *** *p* < 0.01 compared with the HFD group (i.g.). NC, normal control; HFD, high-fat diet; SZ-A, *Ramulus Mori* (Sangzhi) alkaloids; ALT, alanine aminotransferase; AST, aspartate aminotransferase; CRP, C-reactive protein; H&E, hematoxylin and eosin staining; TNFα, tumor necrosis factor alpha; MCP1, monocyte chemoattractant protein-1; Lgalsl1, lectin, galactoside-binding, soluble, 1; Lgalsl3, lectin, galactoside-binding, soluble, 3; Col1a1, collagen type I alpha 1 chain, Col1a2, collagen type I alpha 2 chain, Col3a1, collagen type III alpha 1 chain; Col6a3, collagen type VI alpha 3 chain; Timp1, tissue inhibitors of metalloproteinase-1, Vim, vimentin; Mmp2, matrix metallopeptidase 2; Mmp9, matrix metallopeptidase 9.

NAFLD is characterized by lipid overload, oxidative stress-induced cellular damage, inflammation, and fibrosis. Liver weight and serum alanine aminotransferase (ALT), aspartate aminotransferase (AST), and C-reactive protein (CRP) levels were significantly elevated in mice administered an HFD diet. Here, we found that the serum levels of ALT, AST, and CRP were lower in SZ-A-treated mice than in control HFD mice (Figure 3A–C). Liver weight and hepatic triglyceride and total cholesterol levels were significantly reduced by SZ-A (Figure 3D–F). To determine the protective effects of SZ-A on fatty liver, liver sections were examined using hematoxylin and eosin (H&E) and Oil Red O staining. Histological results revealed that liver tissues were damaged by severe steatohepatitis, microvesicular fatty changes, and hypertrophy induced by HFD. SZ-A administration markedly blocked histopathological changes and reduced the number of hepatic lipid droplets (Figure 3G). 

Next, we examined the effects of SZ-A on inflammation and fibrosis. Macrophage markers (F4/80 and CD68) and pro-inflammatory cytokines (tumor necrosis factor alpha (TNFα) and monocyte chemoattractant protein-1 (MCP1)) were significantly upregulated in the livers of HFD-fed mice. However, mRNA expression levels of macrophage markers and cytokines were attenuated in the SZ-A group (Figure 3H). Lectin, galactoside-,binding, soluble 1 (Lgals1) and lectin, galactoside-binding, soluble, 3 (Lgals3), two members of the galectin family that reflect liver damage, were repressed by SZ-A (Figure 3I). Fibrosis-related genes collagen type I alpha 1 chain (Col1a1), collagen type I alpha 2 chain (Col1a2), collagen type III alpha 1 chain (Col3a1), and collagen type VI alpha 3 chain (Col6a3) were upregulated in the liver of the HFD group, and treatment with SZ-A significantly modulated the expression of these genes towards normal levels (Figure 3I). Markers of NASH-like tissue inhibitors of metalloproteinase-1 (Timp1), vimentin (Vim), matrix metallopeptidase 2 (Mmp2), and matrix metallopeptidase 9 (Mmp9) were significantly reduced by SZ-A (Figure 3I). These results suggest that supplementation with SZ-A at a dose of 400 mg/kg/day restored impaired liver function and suppressed inflammation and fibrosis in mice suffering from HFD-induced oxidative toxicity.

### 3.4. Intraperitoneal Administration of SZ-A Protects against Obesity and NAFLD

**Figure 4:**
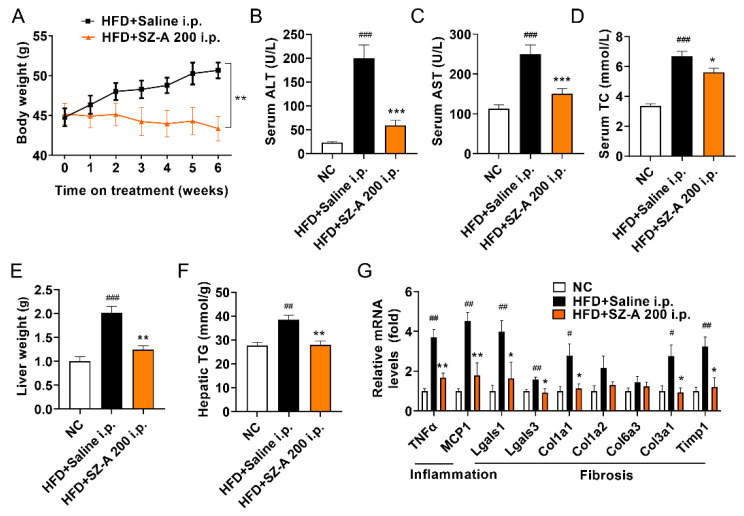
Intraperitoneal administration of SZ-A exerts a protective effect. (**A**) Bodyweight of HFD mice with intraperitoneal administration (i.p.) of saline and SZ-A (200 mg/kg) (*n* = 10/group). Effects of SZ-A on serum levels of ALT (**B**), AST (**C**), and CHO (**D**) are shown. Liver weight (**E**) and hepatic triglyceride levels (**F**) of mice are shown. (**G**) Relative mRNA expression of genes involved in inflammation and fibrosis in livers. Data are represented as mean ± SEM. # *p* < 0.05, ## *p* < 0.01, ### *p* < 0.001 compared with the NC group (i.p.), and * *p* < 0.05, ** *p* < 0.01, *** *p* < 0.001 compared with the HFD group (i.p.). NC, normal control; HFD, high-fat diet; SZ-A, *Ramulus Mori* (Sangzhi) alkaloids; ALT, alanine aminotransferase; AST, aspartate aminotransferase; TC, total cholesterol; TG, triglyceride; TNFα, tumor necrosis factor alpha; MCP1, monocyte chemoattractant protein-1; Lgalsl1, lectin, galactoside-binding, soluble, 1; Lgalsl3, lectin, galactoside-binding, soluble, 3; Col1a1, collagen type I alpha 1 chain, Col1a2, collagen type I alpha 2 chain, Col3a1, collagen type III alpha 1 chain; Col6a3, collagen type VI alpha 3 chain; Timp1, tissue inhibitors of metalloproteinase-1.

To test whether SZ-A exhibited therapeutic effects outside the intestinal tract, HFD-fed mice were administered SZ-A (200 mg/kg) daily via intraperitoneal injection for 6 weeks. Bodyweight was significantly reduced by SZ-A at 200 mg/kg (Figure 4A), which was more effective in bodyweight control than intragastric administration. Serum ALT, AST, and TC levels were lower in SZ-A-treated mice than in HFD mice (Figure 4B–D). In addition, liver weight and hepatic TG levels were significantly decreased by intraperitoneal administration of SZ-A (Figure 4E,F). RT-PCR also showed that intraperitoneal administration of SZ-A decreased the mRNA levels of genes related to inflammation (MCP1 and TNFα) and fibrosis (Lgals1, Lgals3, Col1a1, Col3a1, and Timp1) (Figure 4G). These results show that the protective effects of SZ-A against HFD-induced obesity and NAFLD were independent of the inhibition of α-glucosidase.

### 3.5. Functional Analysis of Differentially Expressed Genes and Pathways in the Liver Tissues of NC, HFD Control, and SZ-A Groups

**Figure 5:**
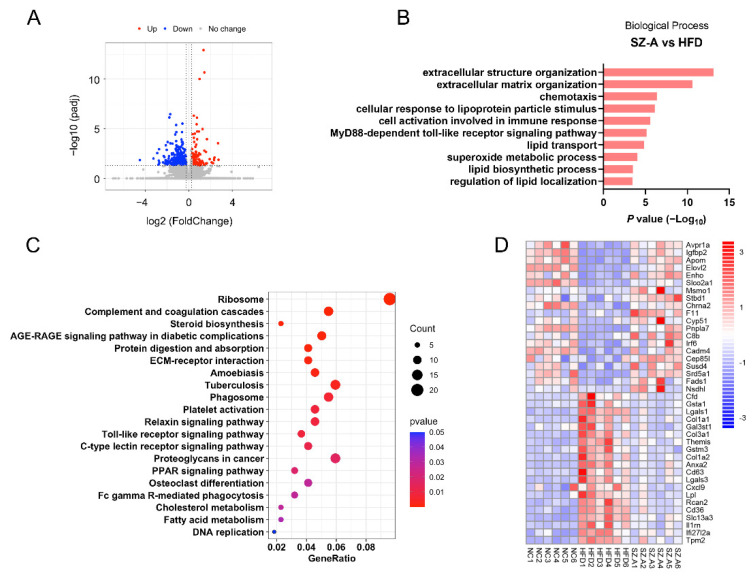
Gene expression signatures of SZ-A-treated mice. (**A**) Volcano plot of the differentially expressed genes (Padj < 0.05, fold change > 1.2) in livers of HFD control and SZ-A group (*n* = 6 per group). Red and blue represent high and low expression of genes in the SZ-A group, respectively. (**B**) GO analysis of biological processes related to NAFLD of differentially expressed genes. (**C**) The 20 highest-ranking terms from KEGG analysis of all differentially expressed genes (Padj < 0.05, fold change > 1.2) are shown. (**D**) Heatmap of the top 20 upregulated and downregulated genes is shown (Padj < 0.05). NC, normal control; HFD, high-fat diet; SZ-A, *Ramulus Mori* (Sangzhi) alkaloids.

To determine the transcriptional changes induced by SZ-A, we performed RNA-seq on the liver tissues of NC, HFD control, and SZ-A-treated HFD mice (*n* = 6 per group). A total of 436 genes were differentially expressed (Padj < 0.05, fold change > 1.2; Figure 5A) between the HFD control and SZ-A group, of which 174 genes were upregulated and 262 were downregulated. To identify the main biological pathways affected by SZ-A, we performed bioinformatic analysis. Gene ontology (GO) enrichment analysis showed that pathways involved in lipid transport and metabolism (cellular response to lipoprotein particle stimulus, lipid transport, lipid biosynthetic process, regulation of lipid localization), oxidative (superoxide metabolic process), inflammation (chemotaxis, cell activation involved in immune response, MyD88-dependent toll-like receptor signaling pathway), and fibrosis (extracellular structure organization, extracellular matrix organization) were significantly affected by SZ-A (Figure 5B). The top 20 pathways enriched from the DEGs in the livers from the HFD control and SZ-A group according to KEGG analysis are shown in Figure 5C. Lipid metabolism pathways including PPAR signaling pathway, cholesterol metabolism, and fatty acid metabolism were enriched. The top 20 upregulated and downregulated genes are showed in Figure 5D. SZ-A treatment significantly decreased mRNA levels of lipid uptake genes (CD36), proinflammatory genes (C-X-C motif chemokine ligand 9 (Cxcl9) and interferon alpha-inducible protein 27-like protein 2A (Ifi27l2a)), and fibrosis genes (Col1a1, Col3a1, and Col1a2) in the liver (Figure 5D). These results revealed that genes and pathways of lipid metabolism and metabolic stress-induced liver injury in mice were regulated by SZ-A.

### 3.6. SZ-A Protects Mice from Oxidative Stress Induced by HFD

**Figure 6:**
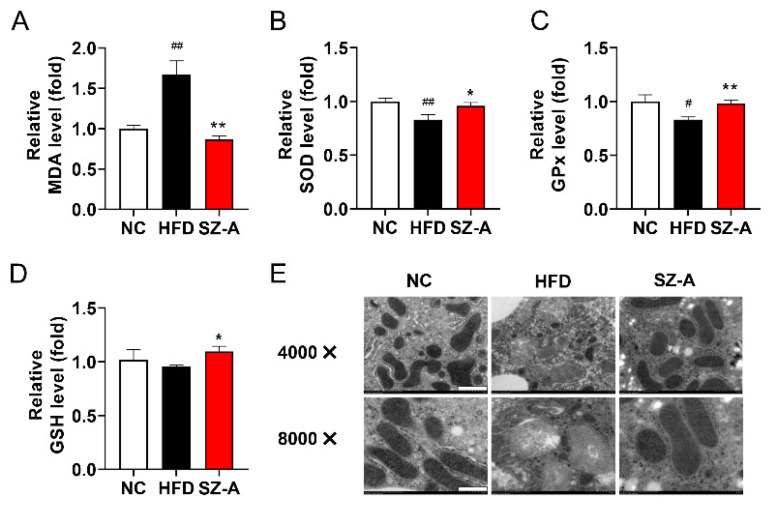
Effect of SZ-A on HFD-induced oxidative stress and mitochondrial damage. (**A**–**D**) Effects of SZ-A on liver MDA levels, SOD activity, GSH levels, and GPx activity (*n* = 6/group). (**E**) TEM analysis of mitochondrial structure in 4000× (scale bar 1 μm) and 8000× (scale bar 0.5 μm) magnifications (*n* = 3/group). Data are represented as mean ± SEM. # *p* < 0.05, ## *p* < 0.01 compared with the NC mice (i.g.), and * *p* < 0.05, ** *p* < 0.01 compared with the HFD mice (i.g.). NC, normal control; HFD, high-fat diet; SZ-A, *Ramulus Mori* (Sangzhi) alkaloids; MDA, malondialdehyde; SOD, superoxide dismutase; GSH, glutathione; GPx, glutathione peroxidase.

Oxidative stress stimulated by lipid overload is harmful to cells. MDA is a biomarker of lipid peroxidation. Cellular MDA levels reflect the degree of oxidative stress. We found that hepatic MDA in SZ-A-treated mice was lower than that of the HFD control group (Figure 6A). To evaluate the superoxide metabolic process, the functions of liver antioxidants in HFD-fed mice were measured. In the HFD group, the levels of SOD, GSH, and GPx were significantly downregulated compared with those in the control group (Figure 6B–D). SZ-A treatment restored the antioxidant capacity of the liver to normal levels. The homeostasis of energy metabolism is mainly regulated by mitochondria. Overload of free fatty acid into mitochondria leads to increased ROS production and alteration of mitochondrial functions. Transmission electron microscopy (TEM) analysis of liver sections showed that HFD induced a significant decrease in mitochondrial cristae density and mitochondria swelling. The mitochondrial double membrane structure and cristae damage induced by the HFD diet was ameliorated by SZ-A (Figure 6E). These results showed that SZ-A protected mice form oxidative damage induced by lipid overload.

### 3.7. SZ-A Regulates Fatty Acid Metabolism and Oxidative Stress in the Liver

**Figure 7:**
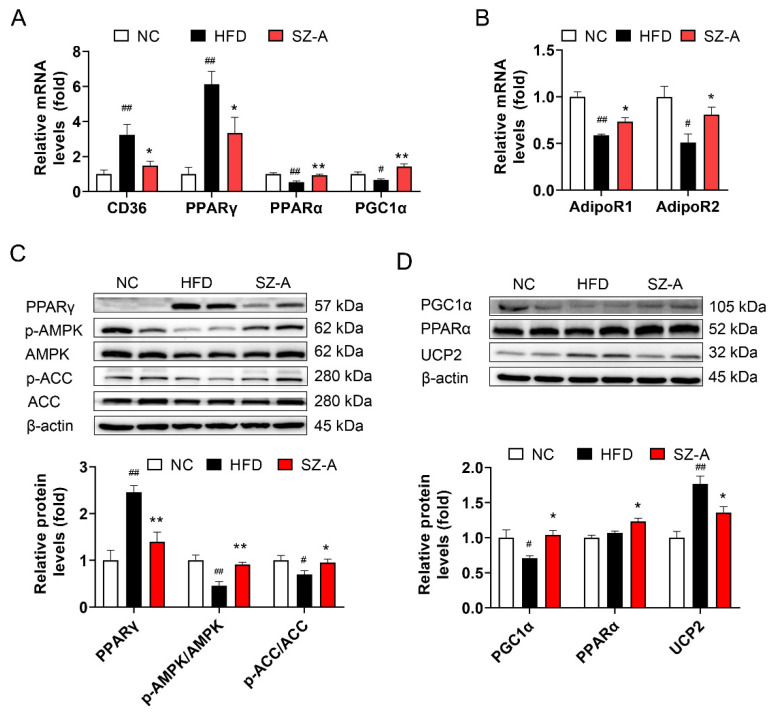
SZ-A regulates lipid metabolism and oxidative stress. (**A**,**B**) Relative mRNA expression levels of genes, including CD36, PPARγ, PPARα, PGC1α, AdipoR1, and AdipoR2. (**C**,**D**) Hepatic PPARγ, p-AMPK, p-ACC, PGC1α, PPARα, and UCP2 protein expression were detected by Western blot with specific antibodies. Data are represented as mean ± SEM (*n* = 6/group). # *p* < 0.05, ## *p* < 0.01 compared with the NC mice (i.g.), and * *p* < 0.05, ** *p* < 0.01 compared with the HFD mice (i.g.). NC, normal control; HFD, high-fat diet; SZ-A, *Ramulus Mori* (Sangzhi) alkaloids; AMPK, AMP-activated protein kinase; ACC, acetyl-CoA carboxylase; p-AMPK, phospho-AMP-activated protein kinase; p-ACC, phospho-acetyl-CoA carboxylase; PPARγ, peroxisome proliferator– activated receptor γ; PPARα, proliferator-activated receptor-α; PGC1α, peroxisome proliferator-activated receptor-γ co-activator 1α; UCP2, uncoupling protein 2.

To further verify the mechanisms of action of SZ-A in hepatic steatosis, the expression of genes involved in lipid production and consumption was evaluated. Quantitative PCR assays showed that the hepatic mRNA levels of genes related to fatty acid uptake and synthesis (CD36 and peroxisome proliferator-activated receptor γ (PPARγ) were significantly diminished after SZ-A treatment. Moreover, the mRNA levels of lipid β-oxidation genes (proliferator-activated receptor-α (PPARα) and PGC1α) were increased in SZ-A-treated mice (Figure 7A). SZ-A intervention also had a positive effect on liver adiponectin receptor expression. In particular, the mRNA levels of adiponectin receptor 1 (AdipoR1) and adiponectin receptor 2 (AdipoR2) were significantly higher in the SZ-A group than in the HFD group, which may mediate adiponectin/AMPK signaling (Figure 7B). Proteins involved in lipid metabolism were also evaluated (Figure 7C,D). The protein levels of the lipogenic gene PPARγ were highly upregulated by HFD, and SZ-A (400 mg/kg) significantly downregulated its expression. AMPK activation reduces NAFLD by regulating de novo lipogenesis, fatty acid oxidation, and promoting mitochondrial function. We found that SZ-A regulated lipid metabolism by increasing the protein levels of p-AMPK and p-ACC. Lipid β-oxidation proteins such as PGC1α and PPARα were markedly higher in the livers of the SZ-A group than those in the HFD group. Besides, SZ-A repressed uncoupling protein 2 (UCP2) expression in protein levels. Our results demonstrate that SZ-A treatment resulted in the activation of AMPK and upregulation of PPARα and PGC1α expression to improve β-oxidation of fatty acids.

### 3.8. SZ-A Regulates Lipid Accumulation and AMPK Signaling in HepG2 Cells Treated with Palmitic Acid

**Figure 8:**
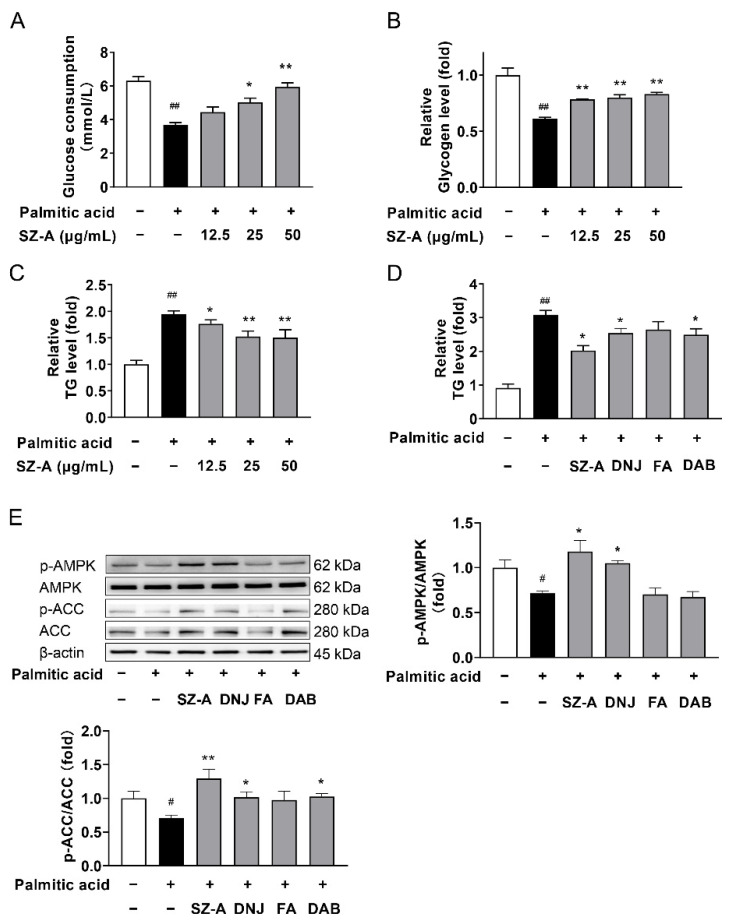
SZ-A regulates the AMPK signaling pathway in HepG2 cells. HepG2 cells were treated with PA (0.125 mmol/L) and/or SZ-A (12.5, 25, or 50 μg/mL), DNJ (40 μg/mL), FA (10 μg/mL), and DAB (10 μg/mL) for 24 h. Glucose consumption (**A**) and glycogen content (**B**) of each group was measured. (**C**,**D**) Levels of TGs in primary hepatocytes in the indicated groups. (**E**) Protein expression levels of p-AMPK, AMPK, p-ACC, ACC, and β-actin in HepG2 cells were analyzed by Western blot. β-Actin was used as an internal reference. Data are shown as the mean ± SEM (*n* = 4/group). # *p* < 0.05, ## *p* < 0.01 compared with cells treated with medium only, and * *p* < 0.05, ** *p* < 0.01 vs. cells treated with PA. TG, triglyceride; SZ-A, *Ramulus Mori* (Sangzhi) alkaloids; DNJ, 1-deoxynojirimycin; DAB, 1,4-dideoxy-1,4-imino-D-arabinitol; FA, fagomine; BSA, bovine serum albumin; PA, palmitic acid; AMPK, AMP-activated protein kinase; ACC, acetyl-CoA carboxylase; p-AMPK, phospho-AMP-activated protein kinase; p-ACC, phospho-acetyl-CoA carboxylase.

To investigate the direct effects of SZ-A on hepatocytes, HepG2 cells stimulated with PA were used as models of insulin resistance and lipid accumulation. PA-stimulated HepG2 cells developed insulin resistance. Compared to the PA group, SZ-A significantly increased glycogen content and glucose consumption in HepG2 cells (Figure 8A,B). We also assessed the inhibitory effects of SZ-A on PA-induced intracellular lipid accumulation in HepG2 cells. As shown in Figure 8C, SZ-A treatment resulted in a dose-dependent reduction in intracellular TG content. The main alkaloids in SZ-A are DNJ, FA, and DAB. The concentrations of the three ingredients were determined according to their proportions in SZ-A. In PA-stimulated HepG2 cells, both DNJ and DAB significantly decreased the intracellular lipid content, whereas the intracellular lipid content of the FA-treated group was inconspicuous (Figure 8D). SZ-A and DNJ markedly increased AMPK and ACC phosphorylation in PA-induced HepG2 cells. In addition, DAB markedly increased the phosphorylation of ACC, but FA had no obvious effect on the ratio of ACC phosphorylation (Figure 8E). These results indicate that SZ-A improved lipid metabolism by activating AMPK and inactivating ACC, which was mainly attributed to DNJ and DAB.

### 3.9. Graphic Illustration of the Mechanism Underlying SZ-A-Mediated Improvement of HFD-Induced Obesity and NAFLD

**Figure 9:**
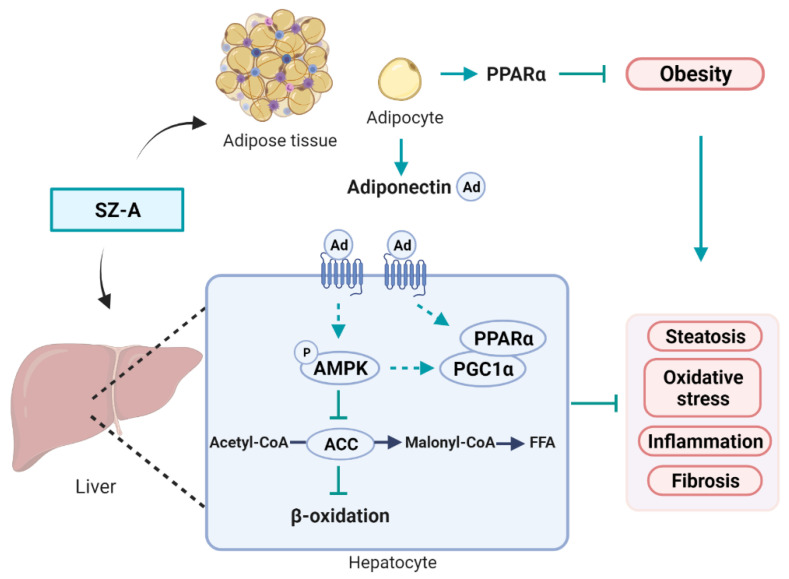
Graphic illustration of the mechanism underlying the SZ-A-mediated improvement of HFD-induced obesity and NAFLD. SZ-A is distributed in the liver and adipose tissue after intragastrical and intraperitoneal administration. SZ-A protects mice from HFD-induced obesity without affecting food consumption. The weight-loss effect contributes partly to the protective effect on NAFLD. Besides, SZ-A may also have direct effects on hepatocytes. AMPK and PPAR signaling pathways are involved in the protective effect. SZ-A, *Ramulus Mori* (Sangzhi) alkaloids; Ad, adiponectin; AMPK, AMP-activated protein kinase; ACC, acetyl-CoA carboxylase; PGC1α, peroxisome proliferator-activated receptor-γ co-activator 1α; PPARα, proliferator-activated receptor-α; FFA, free fatty acid. Dashed lines indicate indirect effect. Created in BioRender.com, accessed on 4 April 2022.

Mice develop obesity and NAFLD when fed with HFD. Excessive lipids accumulate in the adipose tissue, which leads to obesity. Ectopic lipid accumulation in the liver induces oxidative stress and liver injury. Chronic inflammation and fibrosis occur because of metabolic stress. SZ-A is a group of alkaloids that undergo rapid distribution in liver and adipose tissues after administration. The weight-loss effect of SZ-A was partly mediated by activation of PPARα. Reduced fat mass is beneficial for systemic improvement of metabolism. SZ-A stimulates adiponectin expression and secretion, which mediates the crosstalk between liver and adipose tissue. AMPK is activated in the liver and HepG2 cells by direct and indirect effects of SZ-A. The phosphorylation of ACC by p-AMPK inhibits the function of ACC to inhibit lipogenesis and enhance lipid β-oxidation. PPARα and PGC1α are also increased in the liver. In conclusion, SZ-A protects mice from NAFLD through direct effect on hepatocytes and indirect effect mediated by weight loss. AMPK and PPAR signaling are involved in the protective effects of SZ-A (Figure 9).

## 4. Discussion

NAFLD, obesity, and T2DM are common manifestations of metabolic syndrome that shares the same mechanisms, such as hyperlipidemia, insulin resistance, hyperglycemia, and inflammation. Increased hepatic gluconeogenesis promotes insulin resistance and T2DM, further aggravating NAFLD. Excessive fat leads to hypertrophy of adipocyte cells, necrosis, inflammation of adipose tissue, and increased FFA in the liver, which are risk factors for NAFLD [28]. SZ-A, the extract of *Morus alba* L., has been shown to reduce hyperglycemia in patients [24] and was approved for T2DM treatment in 2020, including the regulation of α-glucosidases, insulin sensitivity, microbiota, and inflammation. However, the therapeutic effects of SZ-A on HFD-induced liver injury have not yet been studied.

In the present study, we used mice induced by HFD for 14 weeks as a model, followed by administration of SZ-A for another 6 weeks to study the therapeutic effect of SZ-A. We found that SZ-A ameliorated HFD-induced obesity and inhibited the increase in serum total cholesterol levels. Liver FFAs are derived from adipose tissue lipolysis, de novo lipogenesis, and diet. The fates of FFAs are β-oxidation and triglycerides formation. Excessive FFAs in the liver induce lipotoxicity when their supply and disposal are disturbed [14]. We found that SZ-A inhibited CD36 and PPARγ mRNA expression and promoted PPARα mRNA expression, suggesting that SZ-A may decrease hepatic lipid accumulation and steatosis by inhibiting de novo lipogenesis and fatty acid uptake, while promoting β-oxidation. Excessive fatty acids are toxic to hepatocytes by inducing inflammation and, ultimately, fibrosis. In our study, mRNA expression of inflammatory markers such as TNFα, MCP1, F4/80, and CD68 was restrained by SZ-A. Additionally, genes associated with collagen production, such as Col1a1, Col3a1, and Timp1, were downregulated by SZ-A. These results show that SZ-A has therapeutic effects on NAFLD in mice by inhibiting hepatic steatosis, inflammation, and fibrosis. 

To further investigate the target tissues that mediate the protective effect of SZ-A on obesity and NAFLD, SZ-A was intraperitoneally administered to HFD-induced mice for 6 weeks. We found that the bodyweight of mice treated with SZ-A was significantly lower than that of mice in the control group. Serum ALT and AST levels, hepatic steatosis, inflammation, and fibrosis were also reduced. These results imply that SZ-A may exert a protective effect on organs other than the intestinal tract. It has been reported that the major distribution tissues of DNJ, FA, and DAB are the gastrointestinal tract, liver, and kidney, which provide a rational basis for the direct effect in these tissues [25].

Here, we focused on the direct effects of SZ-A and its major constituents, DNJ, FA, and DAB, on hepatocytes and adipocytes. PA induces lipid accumulation and insulin resistance in hepatocytes. In vitro studies revealed that SZ-A alleviated PA-induced lipid accumulation in HepG2 cells, indicating a direct effect of SZ-A on hepatocytes. It is mainly DNJ and DAB that play a role in activating the p-AMPK/p-ACC pathway and lowering liver triglycerides and cholesterol. SZ-A and its different constituents were added during the differentiation of 3T3-L1 adipocytes. We found that SZ-A promoted adiponectin secretion in 3T3-L1 adipocytes. DNJ and DAB are major constituents that stimulate adiponectin secretion. In terms of its anti-inflammatory effects, it has been reported that DNJ and FA are effective in inhibiting supernatant IL-6, whereas DAB is more effective in suppressing TNFα [23]. These results suggest that the different components of SZ-A may exert different synergistic effects. 

Obesity is an important pathogenic factor for NAFLD and T2DM. Agents with mechanisms of insulin sensitization and bodyweight loss have been studied for NAFLD treatment. It is reported that treatment resulting in a 5% weight loss decreases hepatic fat content [29]. The effect of GLP1 receptor agonists on NASH endpoint are thought to be associated with weight loss and systemic improvements in metabolism [4]. In our study, the bodyweight of SZ-A-treated mice was significantly decreased compared with the HFD control group. The decreased bodyweight contributes partly to reduced fatty liver. However, food consumption was not affected. To elucidate the mechanism of reduced fat mass, we found that PPARα, a nuclear receptor important for lipid metabolism, is increased by SZ-A treatment. SZ-A promotes fatty acid oxidation in adipose tissue of mice.

Adipose tissue plays an important role in systemic nutrient and energy homeostasis. Intercellular and interorgan communication is mediated by adipokines secreted by adipocytes. Adiponectin has been shown to inhibit inflammation, suppress oxidative stress, and accelerate fatty acid oxidation by binding to adiponectin receptors [30]. The synthesis of bioactive adiponectin is difficult, whereas agents that stimulate the secretion of adiponectin are promising [31]. In this study, we measured the effects of SZ-A and its major components on adiponectin expression both in vivo and in vitro. SZ-A promoted adiponectin secretion in 3T3-L1 adipocytes via its components DNJ and DAB. The protein levels of adiponectin also increased in the adipose tissue of SZ-A-treated mice. Increased adiponectin in circulation binds to adiponectin receptors on hepatocytes and exerts protective effects on lipid metabolism and inflammation. In the liver, adiponectin binds with AdipoR1 to activate AMPK pathways and AdipoR2 to activate PPARα pathways [32]. The anti-lipotoxic effects of adiponectin/AdipoR may also be mediated by enhanced activity of PPARα and PGC1α [33].

Lipid overload and oxidative stress-induced cellular damage are important factors in NAFLD pathogenesis. Hepatic lipid accumulation is the result of a disequilibrium between lipid production and consumption. AMPK is a master regulator of cellular energy homeostasis [34]. Phosphorylation of ACC by p-AMPK inhibits lipogenesis and enhances β-oxidation [35]. In this study, we found that SZ-A administration increased p-AMPK (Thr172) and p-ACC levels in SZ-A-treated HFD mice and HepG2 cells, which resulted in decreased lipogenesis and increased fatty acid oxidation. Activation of AMPK regulates PGC1α activation, and activation of Ca^2+^/calmodulin-dependent protein kinase regulates PGC1α expression [36]. PGC1α is a coactivator of many transcription factors, including mitochondrial transcription factor A (TFAM) and nuclear respiratory factors (nuclear respiratory factor 1 (NRF1) and nuclear respiratory factor 2 (NRF2)) to regulate mitochondrial biogenesis, gluconeogenesis, and fatty acid β-oxidation [37]. We found that SZ-A upregulated the expression of PGC1α at mRNA and protein levels. UCP2 is a member of the anion carrier superfamily in the mitochondrial inner membrane. It has been reported that obesity-induced UCP2 expression in hepatocytes promotes hepatic ATP depletion and causes acute liver injury [38]. We found that SZ-A decreased liver UCP2 expression at the protein level to protect the mice from liver injury. These results are in accordance with the morphological changes of mitochondria.

Oxidative stress reflects the imbalance between ROS production and scavenging [39]. ROS causes oxidative modifications to cellular macromolecules, leading to damage to macromolecules and liver injury. MDA is a lipid oxidation product of ROS. Hepatic lipid overload affects different ROS generators, including the mitochondria, endoplasmic reticulum, and NADPH oxidase. Enzymatic antioxidants such as SOD, glutathione peroxidase (GPx), catalase, and nonenzymatic antioxidants like glutathione (GSH) help to maintain a steady level of ROS [40]. The regulation of the antioxidant system has emerged as an interesting target for NAFLD treatment [41]. We found that HFD feeding led to reduced SOD, GPx, and GSH levels in the liver of NAFLD mice, which are major antioxidant biomarkers [42]. These effects were significantly restored by SZ-A treatment, suggesting that SZ-A may enhance ROS scavenging ability. These results demonstrate that SZ-A increased antioxidants to remove HFD-induced toxic oxidative stress in mice.

Our study has some limitations. The protective effect of SZ-A on NAFLD cannot be separated from its weight-loss effect in this study. More studies are needed to compare the effect of SZ-A to food restriction resulting in the same weight loss on HFD-induced NAFLD. The mechanism of bodyweight loss is not clear, though we found that oxidation of fatty acid was influenced by SZ-A administration in mice. Mouse models such as methionine-choline deficient (MCD) diet-induced NASH should also be tested for effects of SZ-A in mice. We have tested the effect of SZ-A on HepG2 cells in vitro. However, the effect of SZ-A on hepatic stellate cells requires further investigation. Besides, the direct target and mechanism of individual components of SZ-A remains unknown, though the effects of SZ-A on adipocyte adiponectin expression and hepatocyte triglyceride metabolism were reported. 

## 5. Conclusions

In this study, HFD-induced obesity, hepatic steatosis, oxidative stress, inflammation, and fibrosis in mice were ameliorated by SZ-A administration. We speculate that SZ-A may act through multiple pathways. PPARα contributes partly to reduced bodyweight. SZ-A attenuates NAFLD by both weight loss and direct effects on HepG2 hepatocytes. AMPK and PPAR signaling pathways play important roles in the protective effects of SZ-A. In summary, the present study in mice revealed that SZ-A is a promising agent for the treatment of complex diseases, such as obesity and NAFLD.

## Data Availability

RNA sequencing data have been submitted to the Gene Expression Omnibus (GEO) under accession number (GSE199105).

## Supplementary Materials

The following supporting information can be downloaded at: https://www.mdpi.com/article/10.3390/antiox11050905/s1, Table S1: Primers for RT-PCR. Figure S1: The major alkaloids in SZ-A determined using HPLC-MS. Miglitol is the internal standard.
