# Supplementary material for: Ramulus Mori (Sangzhi) Alkaloids Alleviate High-Fat Diet-Induced Obesity and Nonalcoholic Fatty Liver Disease in Mice
Source: Antioxidants (Basel). 2022 May 5;11(5):905. doi: 10.3390/antiox11050905 (PMC9137915; doi:10.3390/antiox11050905)
Supplement: Supplementary file 1 [file antioxidants-11-00905-s001.zip › antioxidants-1684435-SI.pdf]

Ramulus Mori (Sangzhi) alkaloids (SZ-A) alleviate high-fat diet-induced obesity and nonalcoholic fatty liver disease in mice

SUPPLEMENTARY FIGURES

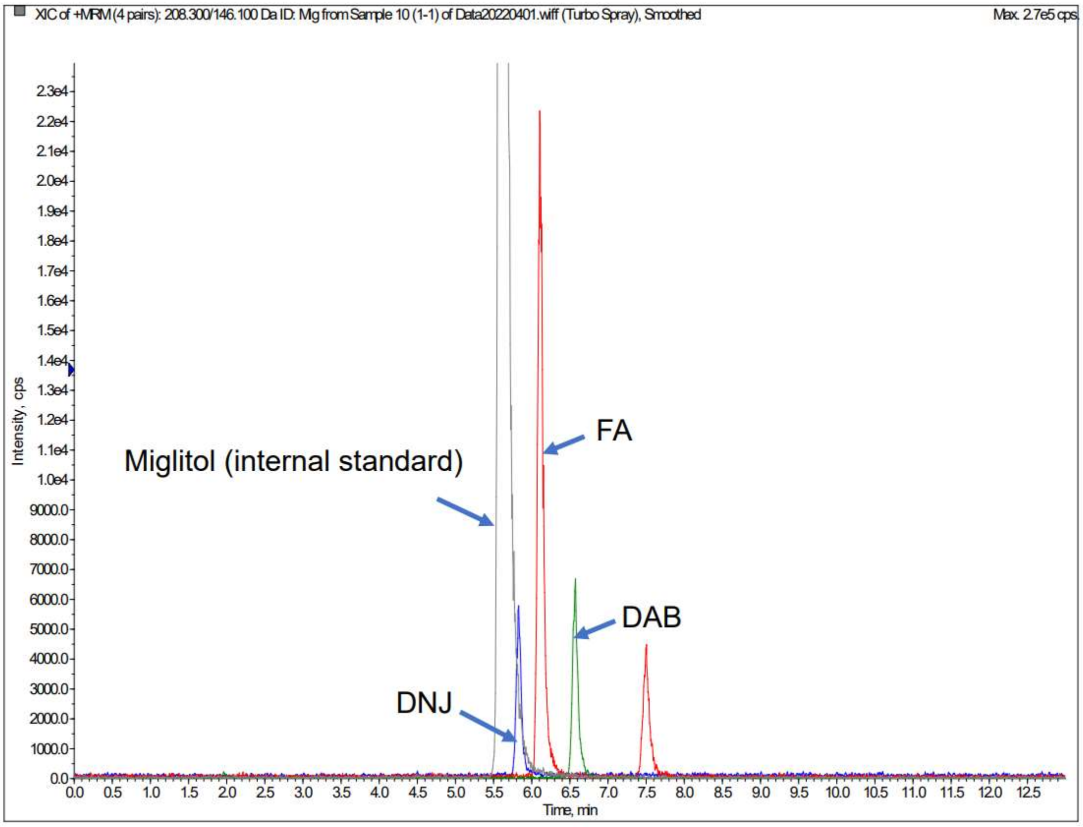

Figure S1. The major alkaloids in SZ-A determined using HPLC-MS. Miglitol is the internal standard.
